# ShinyDegSEM: an interactive application for pathway perturbation analysis in gene expression studies *via* structural equation modeling

**DOI:** 10.7717/peerj.20033

**Published:** 2025-10-10

**Authors:** Zhehan Jiang, Jihong Zhang, Yuanfang Liu, Jinying Ouyang, Linlin Sun, Hao Guo

**Affiliations:** 1 Institute of Medical Education, Peking University Health Science Center, Beijing, China; 2 National Center for Health Professions Education Development, Peking University, Beijing, China; 3 Department of Counseling, Leadership, and Research Methods, University of Arkansas at Fayetteville, Fayetteville, AR, United States of America; 4 School of Public Health, Peking University, Beijing, China; 5 Department of Neurobiology, School of Basic Medical Sciences, Peking University, Beijing, China; 6 School of Health Humanities, Peking University, Beijing, China

**Keywords:** Structural equation modeling, Shiny, Differentially expressed genes, Significance analysis of microarray, Perturbed pathway analysis

## Abstract

**Background:**

Researchers in biology and bioinformatics are increasingly interested in unraveling the complex mechanisms underlying phenotypic variations. A key challenge lies in identifying perturbed biological pathways and understanding how these perturbations propagate through intricate gene regulatory networks.

**Results:**

To address this challenge, we developed ShinyDegSEM, an interactive R Shiny application that leverages structural equation modeling (SEM) to facilitate pathway perturbation analysis in gene expression studies. ShinyDegSEM streamlines identifying differentially expressed genes (DEGs), generating pathway models based on biological knowledge, and evaluating these models to uncover perturbed pathway modules. This article is a tutorial to guide users through the analysis workflow, providing detailed explanations and examples. This feature ensures that even novice researchers can quickly grasp the concepts and apply the tool to their datasets.

**Conclusions:**

The application integrates multiple steps, including DEG detection using significance analysis of microarray, perturbed pathway analysis with signaling pathway impact analysis, and SEM-based model refinement and comparison between experimental and control groups. The interactive interface of ShinyDegSEM allows researchers to easily upload their gene expression data, select appropriate criteria for DEG detection and pathway analysis, and visualize the results in intuitive graphs and tables. The tool provides insights into deregulated genes and modified gene-gene relationships within perturbed pathways.

## Introduction

Biological networks have been popular in recent years (Chin et al., 2014; Omony, 2014; Liu et al., 2020; Wang et al., 2021), stemming from recognizing that biological systems are inherently complex, with numerous interconnected components operating in concert to maintain cellular homeostasis and adapt to environmental stimuli (Goldstein, 2019; Liu et al., 2020). Network biology employs graph-theoretic approaches to represent biological molecules, such as genes, proteins, and metabolites, as nodes in networks, where edges represent the interactions among these components (Alm & Arkin, 2003; Albert, 2007). This paradigm shift has not only enhanced our understanding of biological processes but has also provided a new platform for various applications of analytical frameworks and tools such as machine learning (Muzio, O’Bray & Borgwardt, 2021), statistical modeling (Lee & Tzou, 2009; Oates & Mukherjee, 2012; Epskamp, Rhemtulla & Borsboom, 2017; Valdeolivas et al., 2018), and pathway analysis (Isci et al., 2011; Rodchenkov et al., 2019). These tools enable researchers to unravel the complexities of biological networks, predict behaviors, and identify potential intervention points (Lee & Tzou, 2009).

### Structural equation modeling (SEM) framework

Among the analytical frameworks, structural equation modeling (SEM; Kline, 2023) stands out due to its unique capability to handle complex relationships in measurement models and between latent variables. SEM is a statistical method that allows researchers to test complex theories by examining the relationships between multiple variables (Anderson & Gerbing, 1988; Ullman & Bentler, 2013; Kline, 2023). Specifically, SEM combines factor analysis, multiple regression, and path analysis. SEM allows researchers to build and evaluate models that demonstrate how various variables are connected and influence one another. The mathematical expressions and notations (Pepe & Grassi, 2014; Kline, 2023) are in the Supplementary Materials (SM).

#### SEM in biological studies

Conventional SEM uses measurement and structural models to examine the relationships between observed and latent variables. The SEM method in this paper focuses on relationships between observed variables (*e.g.*, gene expression) while accounting for unobserved factors and using path diagrams to visually represent the models. This approach is well-suited for analyzing gene expression data and uncovering the underlying mechanisms of biological pathways (Liu, dela Fuente & Hoeschele, 2008; Neto et al., 2010; Cai, Bazerque & Giannakis, 2013; Romdhani et al., 2015; Wang, Lu & Miao, 2016; Igolkina et al., 2018).

Researchers have applied SEM in biological and health studies, especially with biological network techniques (Liu, dela Fuente & Hoeschele, 2008; Neto et al., 2010; Cai, Bazerque & Giannakis, 2013; Romdhani et al., 2015; Wang, Lu & Miao, 2016; Igolkina et al., 2018). For example, Liu, dela Fuente & Hoeschele (2008) examined using linear SEM to identify sparse networks, validating genetic network inference through simulation and application to real genetic datasets. The researchers found that SEM was promising for accurately identifying different network edges. Neto et al. (2010) developed a quantitative trait loci (QTL)-driven phenotype network method called QTLnet to jointly infer causal networks and the genetic architecture of sets of phenotypes. They validated this framework through simulations and real data analysis. The QTLnet method incorporates SEM features, using graphical models to illustrate causal relationships between genes and phenotypes and within phenotypes. Likewise, Romdhani et al. (2015) proposed a test to analyze the relationships between genetic variants of gene candidates and correlated traits. They applied this method to real data to examine associations between genes and cardiovascular disease-related traits. Their approach leverages SEM to model complex relationships, providing a robust framework for understanding how genetic variants influence multiple correlated traits simultaneously. Cai, Bazerque & Giannakis (2013) contributed to developing a sparsity-aware maximum likelihood (SML) algorithm for using sparse structural equation models to model gene regulatory networks. Similarly, Wang, Lu & Miao (2016) proposed an efficient structural identifiability analysis algorithm for static linear SEM to help examine graphical models of biological networks with latent variables. In addition, Igolkina et al. (2018) applied the SEM to assess differences in gene expression pathway coefficients between gene network data from 144 schizophrenia (SCZ) patients and 111 control individuals (without SCZ themselves and no family history of SCZ). They found that the SEM can identify the altered relationships between gene interactions at different statistical significance levels (*e.g.*, *p* < .01).

Moreover, various R packages that can apply SEM in biology studies have been developed, such as *GenomicSEM* (Grotzinger et al., 2019), *GW-SEM* (Pritikin et al., 2021), *SEMgraph* (Grassi, Palluzzi & Tarantino, 2022; Grassi & Tarantino, 2025a), *SEMdeep* (Grassi & Tarantino, 2025b).

For example, the *SEMgraph* package facilitates causal network analysis *via* SEM, enabling hypothesis-driven testing of edge-specific effects and group differences in biological systems (*e.g.*, gene regulatory networks). Its framework supports both confirmatory (theory-driven) and exploratory (data-driven) modeling, making it suitable for identifying context-dependent pathway disruptions in diseases (Grassi, Palluzzi & Tarantino, 2022).

Literature shows that although SEM has shown great promise in the biological and health field, its full potential in applied research remains untapped, mainly due to the relatively low collaboration between SEM methodologists and biological researchers. This gap can be attributed to several factors, including the technical complexity of SEM, the distinct backgrounds and terminologies used by researchers from different fields, and the limited exposure of biological researchers to SEM methodologies.

#### Advantages of SEM in pathway analysis

**Hypothesized Causal Relationships *via* SEM**. Structural equation modeling enhances pathway analysis by addressing the critical limitations of traditional correlation-based methods. Unlike approaches that only identify correlated relationships, SEM evaluates hypothesized causal structures, modeling both direct and indirect regulatory influences (*e.g.*, gene *A* → gene *B* → gene *C*). This allows researchers to test mechanistic explanations for observed gene expression changes, such as cascading effects or feedback loops.

In genetic pathway analysis, SEM uses directed edges (→) to represent regulatory relationships (*e.g.*, transcription factor binding) and bidirected edges (↔) to account for unmeasured confounders (*e.g.*, environmental factors or latent proteins) that jointly affect multiple genes. While initial pathway models (*e.g.*, from the Kyoto Encyclopedia of Genes and Genomes (KEGG; Kanehisa et al., 2002; Kanehisa et al., 2004; Kanehisa et al., 2017) are simplified abstractions of biological networks, SEM provides a framework for validating and iteratively refining these models using empirical data. For example, SEM can test whether adding a hypothesized interaction (*e.g.*, a post-translational modifier) improves model fit, thereby bridging gaps between static pathway maps and dynamic biological reality.

**Comparative Analysis of Regulatory Network Dynamics Across Groups in SEM**. Multiple group analysis in SEM enables comparative evaluation of regulatory interactions involving pre-identified differentially expressed genes (DEGs). By testing invariance in path coefficients and network structures across groups, SEM reveals context-specific rewiring of regulatory relationships, such as strengthened or weakened causal effects between DEGs in disease conditions.

Structural equation modeling extends beyond transcriptomic correlations by testing hypothesized *directed relationships* between genes, even when their RNA levels lack strong pairwise correlations. By modeling pathways (*e.g.*, Gene *B*_1_ → Gene *B*_2_ *via* latent mediators), SEM can infer regulatory effects masked in simple correlation analyses. While SEM cannot directly measure post-translational modifications (PTMs) or dynamic cascades, it can incorporate latent variables to approximate such mechanisms if supported by auxiliary data. The strength of SEM is evaluating how well a predefined network structure (including indirect or hierarchical relationships) explains observed gene expression patterns, revealing path coefficients that reflect hypothesized regulatory influences.

**Multiple Data Sources and Comprehensive Analysis *via* SEM**. The SEM pipeline integrates multi-modal data sources, such as gene expression (microarrays), curated pathway topologies (KEGG), and protein-protein interaction networks (*e.g.*, Search Tool for the Retrieval of Interacting Genes/Proteins (STRING) database Szklarczyk et al., 2015), to construct biologically plausible regulatory models. This integration enhances robustness by cross-validating hypotheses against orthogonal data types. For example, in a study of frontotemporal lobar degeneration with ubiquitinated inclusions (FTLD-U), SEM analysis of the glutamatergic synapse pathway identified *PSD-95* as a hub gene and revealed altered regulatory relationships involving *SHANK2* and glutamate receptors under progranulin mutation. The model further suggested context-specific activation or inhibition of connections (*e.g.*, strengthened *PSD-95* →*SHANK2* interactions in mutant conditions). Similarly, in multiple sclerosis (MS), SEM highlighted dysregulated genes (*ARF6*, *CRKL*, and *PIP5K1C*) within the Fc gamma R-mediated phagocytosis pathway. These findings align with prior studies implicating phagocytic dysfunction in MS pathogenesis (Pepe & Grassi, 2014). SEM disentangles direct regulatory effects from indirect associations, offering mechanistic insights into neurodegenerative processes by combining pathway and interaction data.

#### Model assessment *via* SEM

Structural equation modeling evaluates model fit using statistical tests and indices (Kline, 2023) such as the chi-square test, root mean square error of approximation (RMSEA), and standardized root mean square residual (SRMR). Biological evidence from databases like STRING can be incorporated to validate and include known interactions. A well-fitting model is typically indicated by a non-significant *χ*^2^ test *p* value (though this test is sensitive to sample size), RMSEA ≤ .06 (Hu & Bentler, 1999), and SRMR ≤ .05 or .10 (West, Taylor & Wu, 2012; Grotzinger et al., 2021) for adequate or good fit, respectively. These indices evaluate how closely the proposed model aligns with the observed data. To refine the model, modification indices (MI) estimate the potential improvement in fit (quantified by the expected decrease in *χ*^2^) if a constrained parameter (*e.g.*, a path or covariance) is freely estimated (Kline, 2023). Statistical indices, such as Akaike information criterion (AIC) and Bayesian information criterion (BIC), can also be used for SEM model comparisons and selections (Grassi, Palluzzi & Tarantino, 2022; Kline, 2023). However, modifications are only justified when they align with substantive theory, domain knowledge, or plausible causal mechanisms. Nonsignificant paths may be removed to enhance parsimony if such changes do not compromise theoretical expectations. Iterative adjustments balancing statistical guidance and substantive rationale are critical to avoid overfitting and support generalizability. See the Supplementary Materials (SM) for detailed explanations.

#### Validation for SEM results

Comparative analyses based on other methods, such as simple differential expression or correlation-based network analysis, could be conducted to validate SEM results. Benchmark datasets with known ground truth can validate the accuracy and reliability of SEM. Experimental validation of key SEM findings through assays, such as testing the impact of perturbing specific genes or connections, would confirm predicted changes in gene activity. Evaluating the predictive accuracy of SEM models would also strengthen their assessment (*e.g.*, predicting disease progression or treatment response). Lastly, developing more intuitive visualizations that highlight key findings and show network differences between experimental conditions would enhance the understanding and communication of SEM results. We aim to contribute to the use of SEM for pathway analysis by developing a Shiny application (app).

Interactive biological web applications hosted on Shiny servers have been published more recently due to the increasing awareness among researchers of their methodological advances and practical ease. For example, Jia et al. (2022) systematically reviewed biological web applications built with R or Shiny and their basic and advanced features. However, applications specifically designated to handle SEM are less commonly seen; one of the most well-known is power4SEM, which is used for power calculations (Jak et al., 2021). Our article serves as a tutorial brief to address this gap by developing an R Shiny software application called ShinyDegSEM, which connects bioinformatics with SEM. Although researchers had elegantly applied SEM in gene expression and pathway analysis data (Pepe & Grassi, 2014), to our knowledge, this is the first tool that adopts SEM to investigate perturbed pathway modules derived from gene expression data with interactive visualization. We designed the ShinyDegSEM to make SEM accessible to experimental biologists and computationally inexperienced researchers, while also accelerating analyses for bioinformaticians. The intuitive point-and-click interface integrates complete analytical workflows, enabling complex analyses without programming requirements.

## Materials & Methods

Understanding phenotypic variation requires studying perturbations in complex intracellular networks rather than focusing solely on single-gene dysregulation. High-throughput gene expression data enables investigation of changes in gene expression profiles across different conditions. A comprehensive analysis of pathway perturbation *via* SEM integrates two key components: genome-wide association studies (GWAS) with pathway extensions to identify genetic associations, and SEM-based modeling, evaluation, and refinement to quantify network-level effects. Building on the foundational workflow of Pepe & Grassi (2014), which spans from identifying differentially expressed genes (DEGs) to validating and interpreting perturbed pathway models, we enhance this approach by incorporating recent advancements in GWAS and SEM into ShinyDegSEM. Our implementation offers improved flexibility, usability, and analytical precision for pathway-centric studies. See the SM for detailed gene study terminologies and methodologies. The following steps are needed to apply ShinyDegSEM for conducting pathway analyses using SEM.

**Step 1**. In the initial step, users can collect and prepare data for analysis. Three primary genomic data types can be included: (1) gene expression data (including microarray-based transcript abundance quantification (Schena et al., 1995) and RNA-sequencing (RNA-seq) for genome-wide expression profiling with single-nucleotide resolution (Wang, Gerstein & Snyder, 2009; AlJanahi, Danielsen & Dunbar, 2018), (2) genomic variation data (*e.g.*, whole-genome or exome sequencing data) capturing nucleotide-level polymorphisms and structural variants (DePristo et al., 2011), and (3) quantitative real-time polymerase chain reaction (qRT-PCR) data for precise expression validation (Hendriks-Balk, Michel & Alewijnse, 2007). Public repositories such as NCBI’s Gene Expression Omnibus (GEO; National Center for Biotechnology Information, 2024) and KEGG (Kanehisa et al., 2002; Kanehisa et al., 2004; Kanehisa et al., 2017) may serve as additional data sources. Prepared data (*e.g.*, .txt or .csv formats) are imported for follow-up analysis.

**Step 2.** In Step 2, we identify DEGs to detect significant gene expression level changes between two or more conditions. For microarray data, methods such as significance analysis of microarrays (SAM; Tusher, Tibshirani & Chu, 2001) are commonly employed. RNA-seq data typically utilize count-based approaches, including normalization and statistical modeling *via* negative binomial distributions (Rapaport et al., 2013). Alternative strategies combine fold-change (FC) thresholds with non-stringent *p*-value cutoffs to balance sensitivity and specificity (Shi et al., 2008). Emerging machine learning approaches, including deep learning frameworks, offer additional tools for DEG detection (Tasaki et al., 2020), especially in complex datasets.

**Step 3**. In Step 3, we identify perturbed pathways. Biologically perturbed pathways are identified as functional modules enriched with DEGs, which are indicative of potential disease-associated dysregulation (Pham et al., 2016). Established computational approaches include: (1) enrichment analysis (*e.g.*, over-representation or gene set enrichment; Rahmati et al., 2017), (2) signaling pathway impact analysis (SPIA) that combines topological and statistical metrics (Tarca et al., 2009), and (3) integration with curated pathway databases (*e.g.*, KEGG; Kanehisa et al., 2017). These pathways are subsequently modeled as directed graphs or gene networks, where nodes represent molecular components and edges depict functional interactions, enabling the visualization and topological analysis of perturbed systems (Goh et al., 2007).

Kyoto Encyclopedia of Genes and Genomes (KEGG) pathway graphs were converted into directed graphs for SEM analysis. In this representation, nodes represent genes derived from microarray, RNA-seq data, or Protein Information Resource (PIR) superfamilies, which are clusters of evolutionarily related proteins with shared functions. Edges represent directed biochemical interactions between nodes, which are categorized into two primary types: molecular interactions, including protein-protein binding and enzymatic reactions, and regulatory relationships such as transcriptional activation or suppression (Pepe & Grassi, 2014; Grassi & Tarantino, 2022). The directed graph structure encodes the causal dependencies between molecular components, allowing SEM to quantify pathway-wide dysregulation across comparison groups (or between diseased and normal controls). Main advantages of this approach include: (1) maintaining biological interpretability through preservation of established pathway architectures, (2) enabling quantitative assessment of both magnitude and directionality of molecular interactions, and (3) supporting investigation of condition-specific pathway dysregulation through group comparisons.

Edges can be further classified into two types by directionality (Pepe & Grassi, 2014; Grassi & Tarantino, 2022). Directed edges (→) indicate a direct influence of one gene on another. The direction of the arrow indicates which gene regulates the other. For example, if gene *Y*_1_ has a directed edge pointing to gene *Y*_2_ (*Y*_1_ →*Y*_2_), it means that gene *Y*_1_ is an upstream regulator that directly affects the activity of gene *Y*_2_ (see Fig. S1). Bidirected edges (↔) represent covariances between two genes attributable to unmeasured common causes (*e.g.*, latent upstream regulators or shared environmental factors) influencing both genes (see Fig. S2).

**Figure 1:**
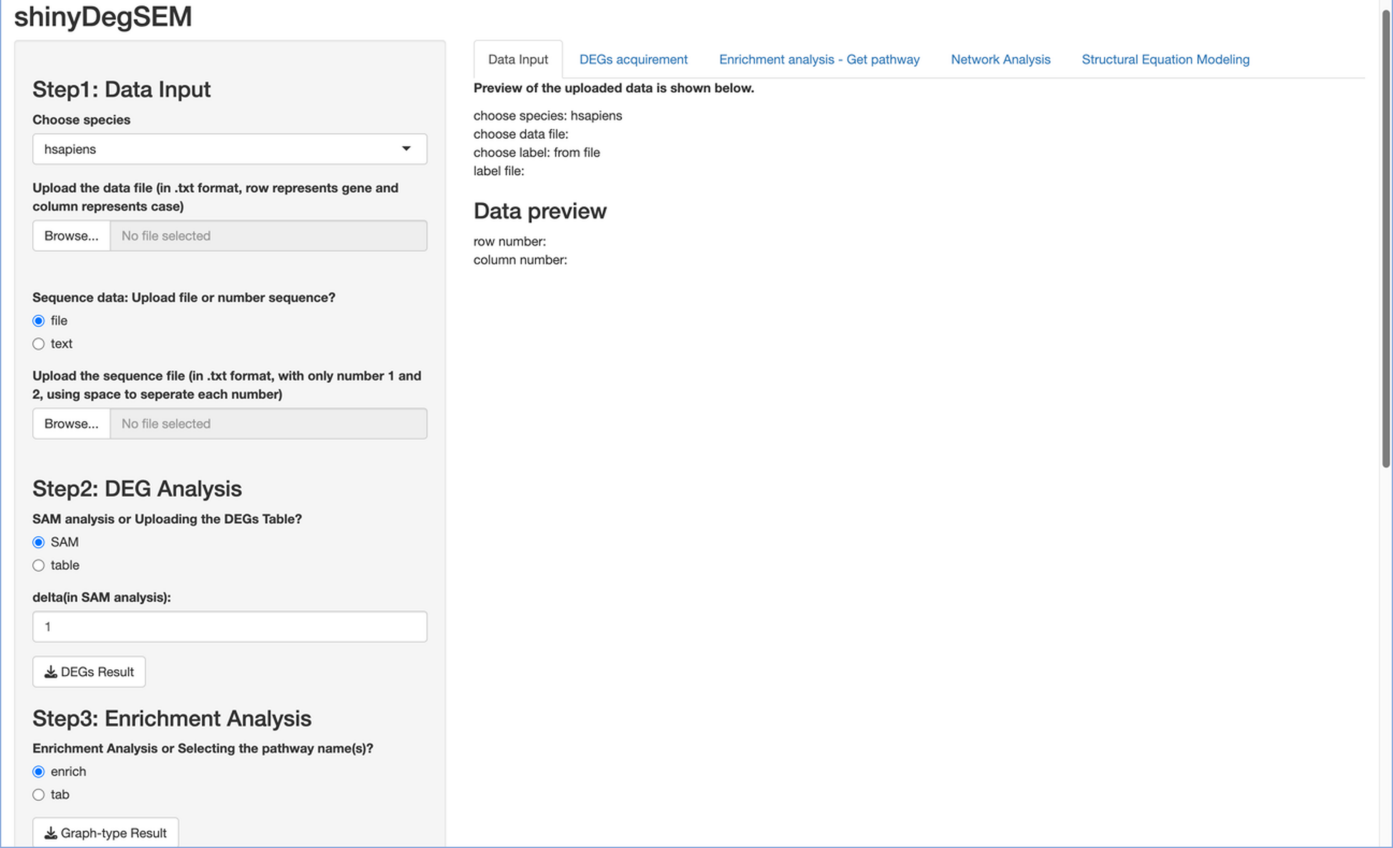
Layout of the ShinyDegSEM application.

**Figure 2:**
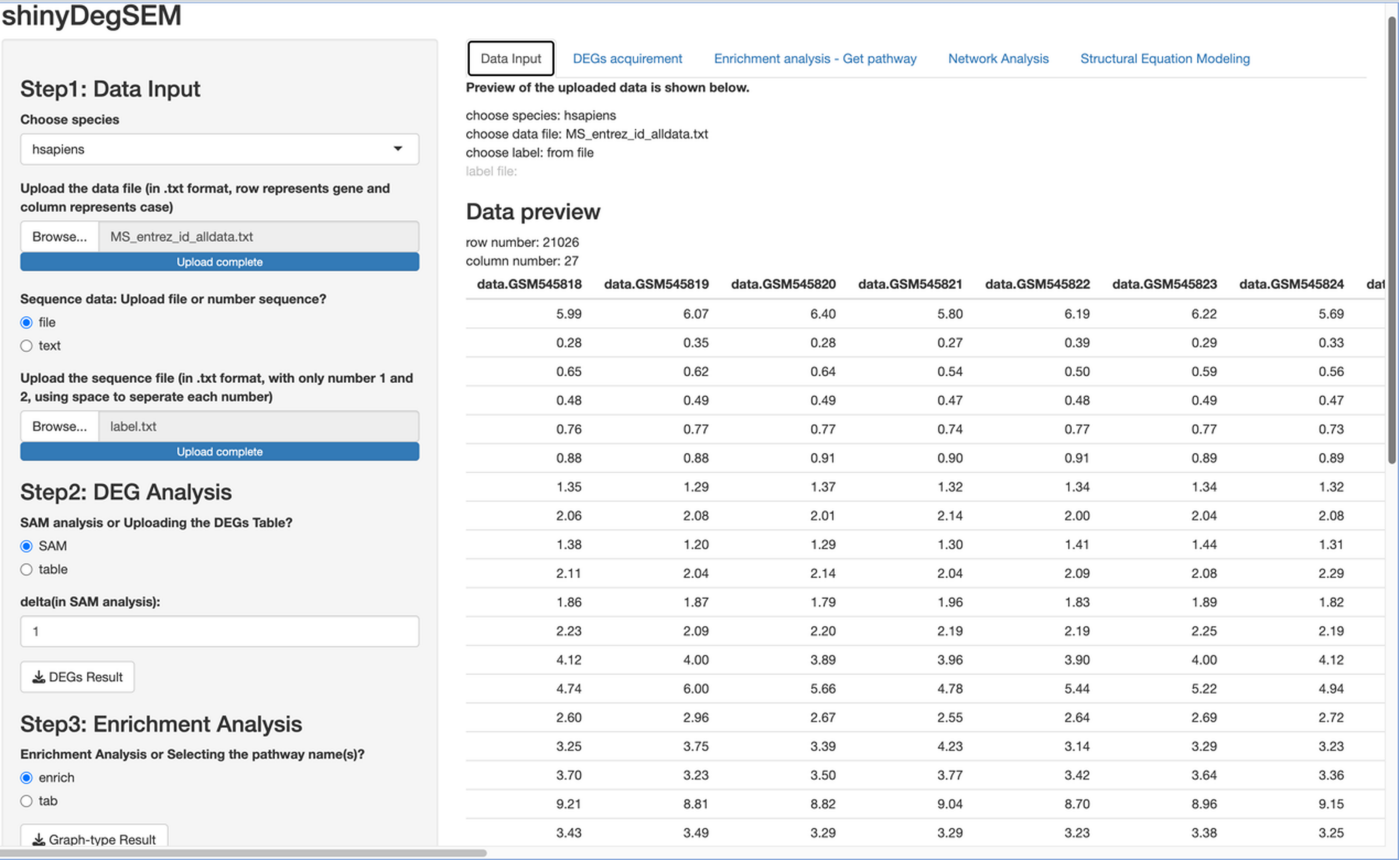
*ShinyDegSEM* interface after data upload. The main panel displays the gene expression matrix (rows: genes; columns: individuals).

These edges in the directed graphs can have signs, which is a crucial aspect of how SEM is used in this context. The strength and direction of the influence between two genes connected by a directed edge (→) are quantified by path coefficients. These coefficients typically range from −1 to 1 if the data are standardized. Positive path coefficients indicate a net activation or positive control, meaning that an increase in the activity of the upstream gene is expected to result in an increase in the activity of the downstream gene. Negative path coefficients represent net inhibition or negative control, meaning that an increase in the activity of the upstream gene is expected to decrease the activity of the downstream gene. On the other hand, bi-directed edges (↔) represent the covariance between two genes *i* and *j* due to unobserved factors, which is quantified by *ψ*_ij_.

Structural equation modeling (SEM) employs linear regression equations in which path coefficients (*β*_ij_) quantify both the strength and direction (*i.e.,* positive or negative) of relationships between variables, serving as weights in the model equations. These signed coefficients are essential for determining the nature of gene-gene interactions within pathways, distinguishing between activating (positive), inhibitory (negative), or latent common-cause relationships. The framework enables comparison of these signed effects across experimental or different conditions through parameter contrasts between groups. During model refinement, MI, *z*-tests, and external biological databases (*e.g.*, STRING; Szklarczyk et al., 2015) can inform the addition of directed or bidirected edges, with database-derived interaction signs directly informing path coefficient directions.

**Steps 4 & 5**. In Step 4, we integrate curated pathway topologies (*e.g.*, from KEGG) with data-driven network filtering using the algorithms proposed by Pepe & Grassi (2014). Canonical pathways are first represented as directed graphs and then pruned using partial correlations derived from gene expression data (*e.g.*, Type I error rates <.05). In Step 5, we apply SEM to the refined pathways, where differential analysis of path coefficients identifies statistically perturbed interactions across groups.

The SEM analyses were conducted using a suite of specialized R packages chosen for their complementary capabilities. Specifically, the ***Lavaan*** package (Rosseel, 2012) served as the primary platform for model specification, parameter estimation, and goodness-of-fit assessment, which provides comprehensive functionality for diverse SEM applications. The *Lavaan* package uses maximum likelihood (ML) estimation by default for continuous and complete data (Rosseel, 2012). For network visualization and manipulation of model components, including latent variable relationships, we employed the *igraph* package (Csardi & Nepusz, 2006; Csárdi et al., 2025), which facilitates intuitive graphical representation and interpretation of complex model structures. Additional analytical support was provided by the *semTools* package (Jorgensen et al., 2022), which offered essential utilities for data diagnostics, model comparison, and advanced statistical evaluations. This package enhanced our analytical workflow through its specialized functions, complementing core SEM procedures. A distinctive aspect of our approach involved integrating network analysis with SEM using the *SEMgraph* package (Grassi, Palluzzi & Tarantino, 2022), which depends on *Lavaan* and *igraph* packages. This specialized tool enabled network-based model exploration, including fitting SEM models, pathway identification, detection of initial nodes, and robustness assessment through graph-theoretic and statistical metrics. Combining traditional SEM with network analysis, *SEMgraph* provided unique insights into model interconnectivity and dynamics.

After estimating the initial SEM model based on the perturbed pathways and gene connections from examined data (*e.g.*, microarray), we obtain the strength of gene-gene connections, also known as path coefficients. The SEM models can be modified based on additional information, such as goodness-of-fit indices (*e.g.*, RMSEA and SRMR), which are used to support the decision on whether to refine them iteratively. After the final structure of the model is determined, the remaining analysis focuses on assessing the appropriateness of group comparison in SEM through invariance tests, examining whether the models differ significantly between groups (*e.g.*, diseased *vs.* healthy), and identifying genes and gene-gene interactions that show significant differences in expression or regulation.

The remaining work is interpretation, where researchers should consider correlating the perturbed genes and connections with known biological processes and disease mechanisms. More importantly, like other biological analyses through statistical mining, it is critical to discuss the implications of the findings to understand the phenotype of interest.

### Shiny walkthrough

#### The layout of the ShinyDegSEM application

We first describe the ShinyDegSEM application (app) layout and then explain how to navigate the main screen. The initial screen of the app is displayed in Fig. 1. The left panel (in gray) includes five steps for user navigation, while the right panel (in white) shows the outputs of each step. The five steps in the app are: (1) Step 1 data input, (2) Step 2 DEG analysis, (3) Step 3 enrichment analysis, (4) Step 4 network analysis, and (5) Step 5 SEM analysis. Specifically, users can click the “*Browse*” button under Step 1 to upload a .txt or .csv data file and start the analysis.

#### Using the ShinyDegSEM application

We used the same gene expression microarray data as Pepe & Grassi (2014) to demonstrate the app’s use. The dataset pertains to MS. It includes genome-wide expression data from peripheral blood mononuclear cells (PBMC) of 12 MS patients and 15 healthy controls, contributed by Kemppinen et al. (2011). The dataset (Kemppinen et al., 2019) is stored in the Gene Expression Omnibus (GEO; National Center for Biotechnology Information, 2024) database under ID GSE21942. Figure 2 shows a screen plot after uploading the dataset and the patient and control group memberships from the label file (in Step 1). The right panel shows a data preview with 21,026 rows for genes and 27 columns for participant IDs. The application can be downloaded from https://osf.io/v9msd/files/osfstorage?view_only=d194b941d7c14f148c618d6532a47a0f. When run, it follows the regular Shiny app execution method.

**Figure 3:**
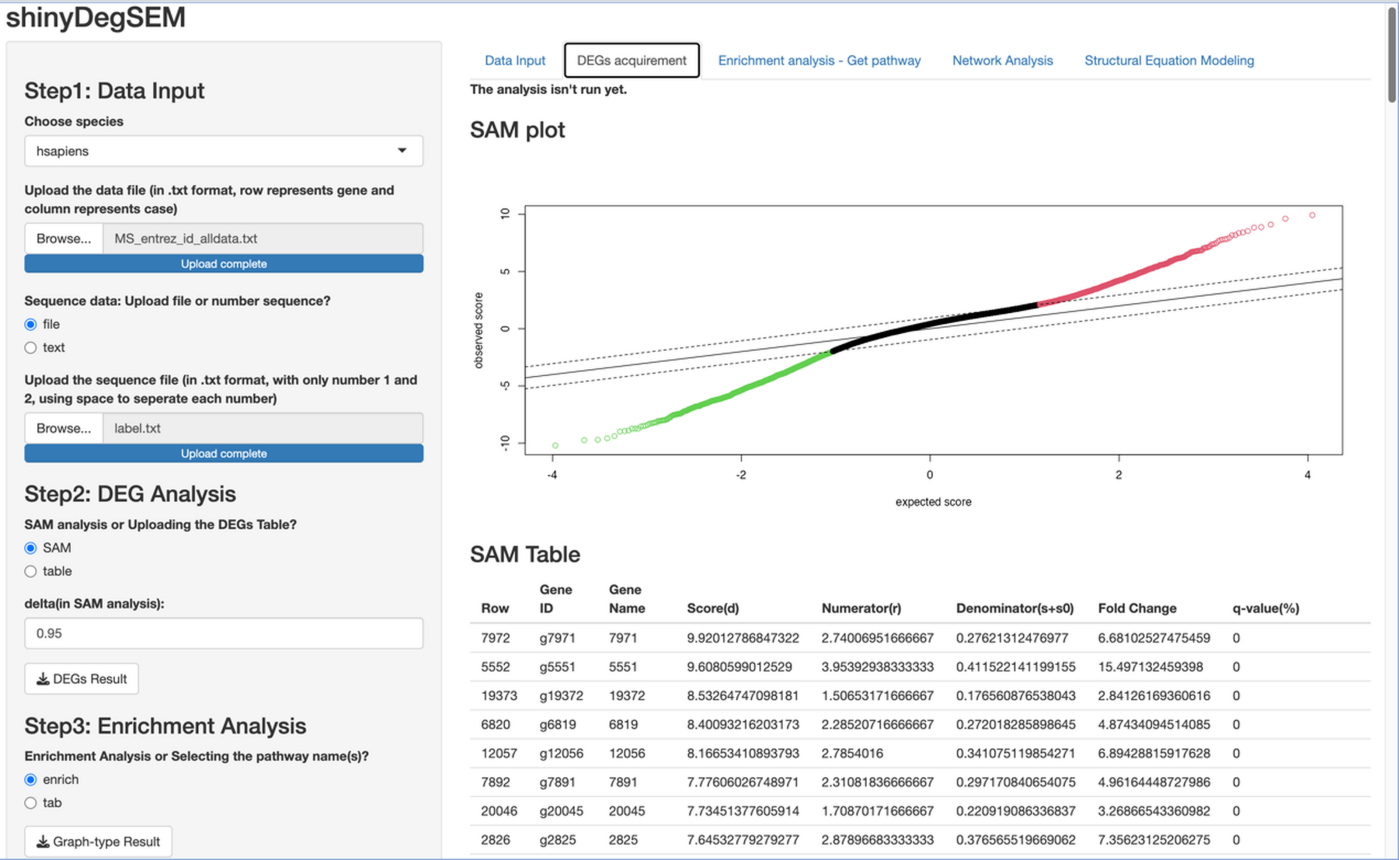
Output of significance analysis of microarrays (SAM) for Multiple Sclerosis (MS) data.

**Figure 4:**
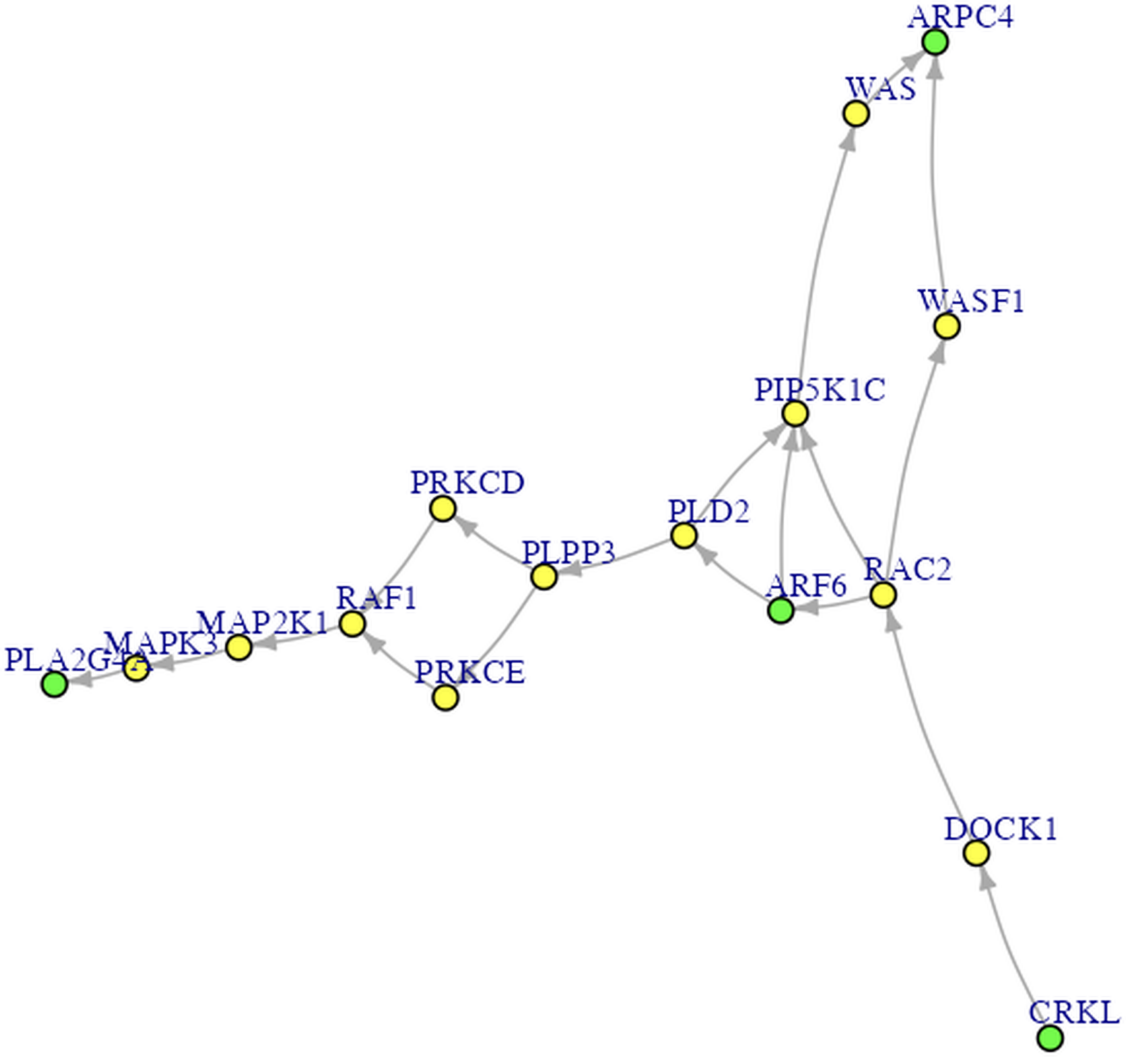
The Fc gamma R-mediated phagocytosis pathway. The green nodes are DEGs. The yellow nodes are not DEGs (*i.e.,* non-DEGs).

**Procedures Before Conducting SEM**. Let’s proceed to Steps 2 through 4 before performing SEM. In Step 2 for DEG analysis, the default delta value (Tusher, Tibshirani & Chu, 2001) in SAM analysis was set to 1 in the app. Specifically, the delta (Δ) threshold is an adjustable parameter that combines both fold-change magnitude and gene-specific variability to balance Type I (*i.e.,* false positive) and Type II (*i.e.,* false negative) error rates in differentially expressed genes (DEGs) analysis (Tusher, Tibshirani & Chu, 2001). Researchers may modify the threshold to emphasize precision or sensitivity based on study objectives. For example, we used 0.95 as Pepe & Grassi (2014) did. Step 3 involved enrichment analysis for identifying perturbed pathways. Step 4 is network analysis. Specifically, Steps 2 and 3 analyses will be performed automatically after uploading the files. Users can click the “*Run Network Analysis*” button to initiate the analysis related to Step 4. After a short wait (depending on the dataset size), results from Steps 2 to 4 will gradually appear in the right panel. For example, clicking the “*DEGs acquirement*” button will display the output of the SAM analysis for DEG analysis (see Fig. 3). Similarly, clicking the “*Enrichment analysis –Get pathway*” button will display the output of different perturbed pathways. By clicking the “*Network Analysis*”, we can see model information and graphs for identified pathways, such as the “B cell receptor signaling”, “Fc gamma R-mediated phagocytosis”, and “Chagas disease” pathways. For example, Fig. 4 shows the identified DEGs and non-differentially expressed genes (non-DEGs) within the context of the Fc gamma R-mediated phagocytosis pathway, which is associated with autoimmune dysregulation and inflammation. The DEGs (*CRKL*, *ARF6*, *PLA2G4A*, and *ARPC4*) were identified (corresponding to Entrez IDs 1399, 382, 5321, and 10093, respectively) and matched those shown in Pepe & Grassi’s (2014) study. Researchers can identify DEGs based on network analysis results and understand the direction of gene interactions within a pathway. These findings can then be incorporated into SEM to investigate causal relationships among more genes and their interactions, providing insights into the regulatory mechanisms underlying the pathway.

## Results

### Running SEM based on network analysis results

**Figure 5:**
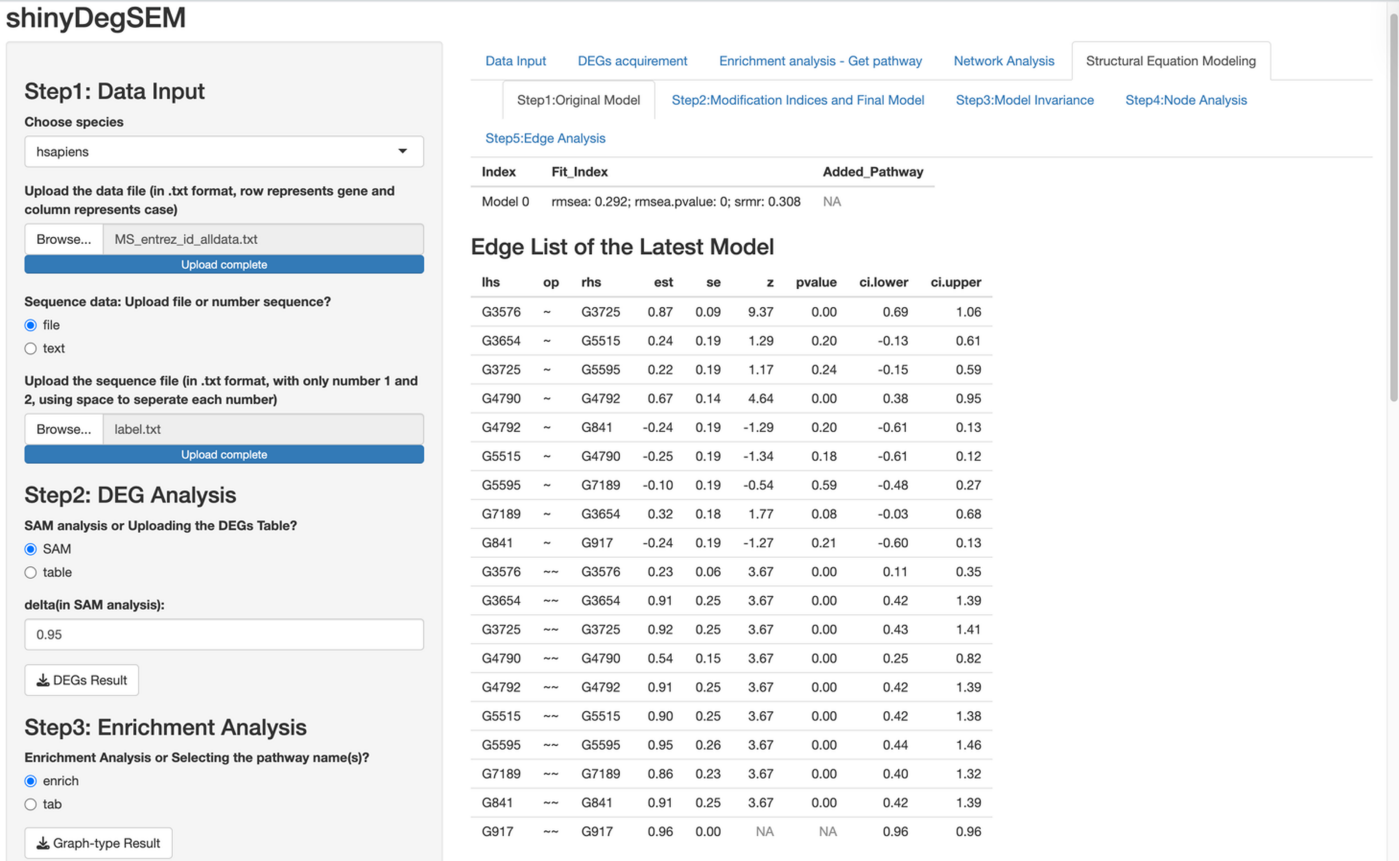
Initial structural equation modeling (SEM) output from network analysis for Chagas Disease pathway.

**Figure 6:**
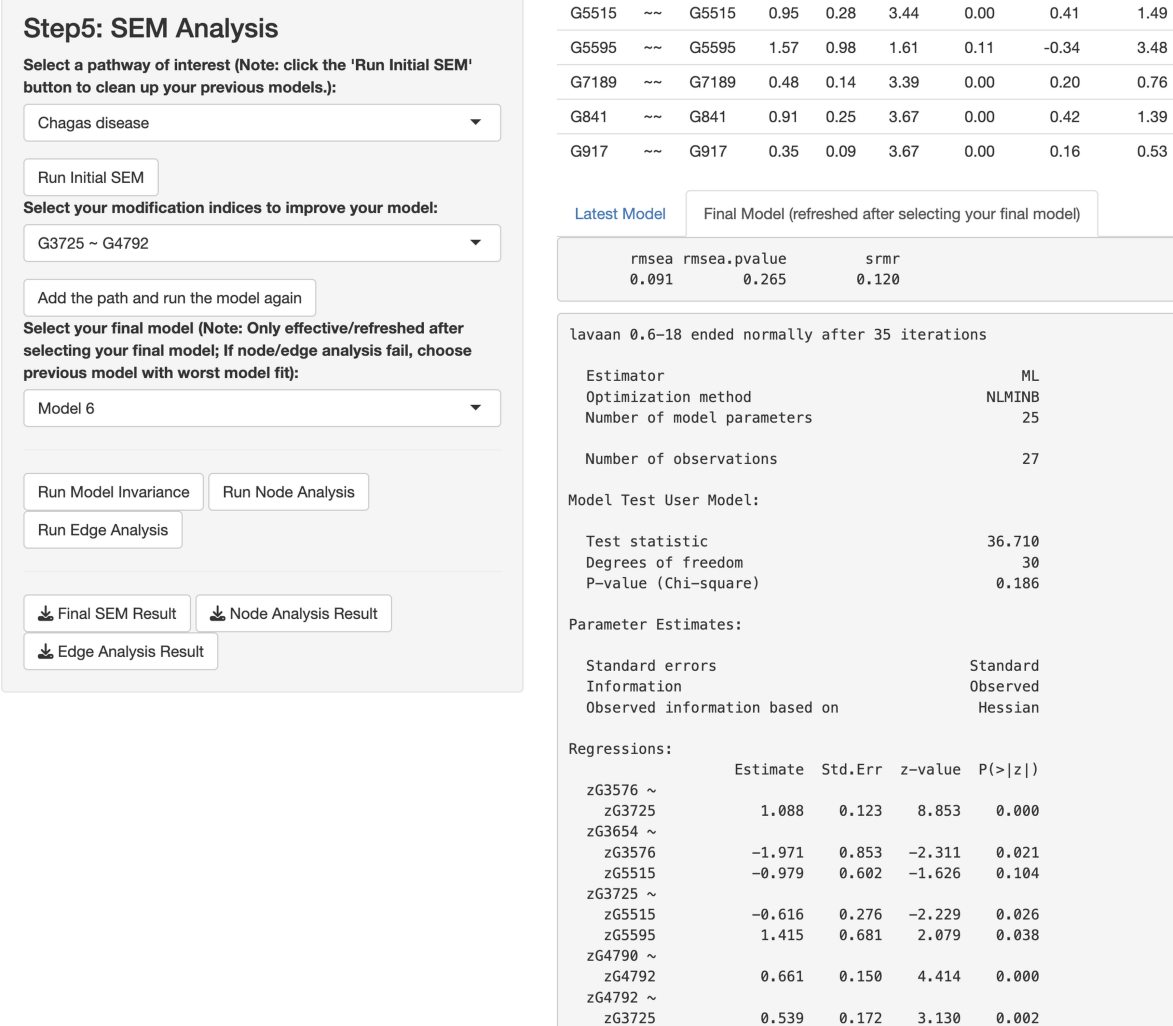
Final structural equation modeling (SEM) output from network analysis for Chagas Disease pathway.

In this paragraph, we describe how to work on SEM based on results from the network analysis and explore gene relationships in Step 5 of the app. First, select one or more pathways of interest from the panel, such as the “Chagas disease” pathway. Second, choose the SEM estimator, which is set to ML by default (Rosseel, 2012), and click “*Run Initial SEM*”. The initial model output will appear in the right panel, displaying the model summary and model fit indices (see Fig. 5), such as the SRMR (Kline, 2023) and the RMSEA (Anderson & Gerbing, 1988). The initial model related to the “Chagas disease” pathway did not fit the data well, with chi-square statistic *χ*^2^(36) = 118.92 and *p* < .001, RMSEA = .292, and SRMR = .308. In addition, we can modify the initial model by selecting an additional path and clicking “*Add the path and run the model again*”. Adding six paths, we improved the model fit substantially (see Fig. 6), with model 6 having chi-square statistic ${\chi }^{2} \left( 30 \right) =36.71$ and *p*= .186, RMSEA = .091, and SRMR = .120.

#### Invariance evaluation

**Figure 7:**
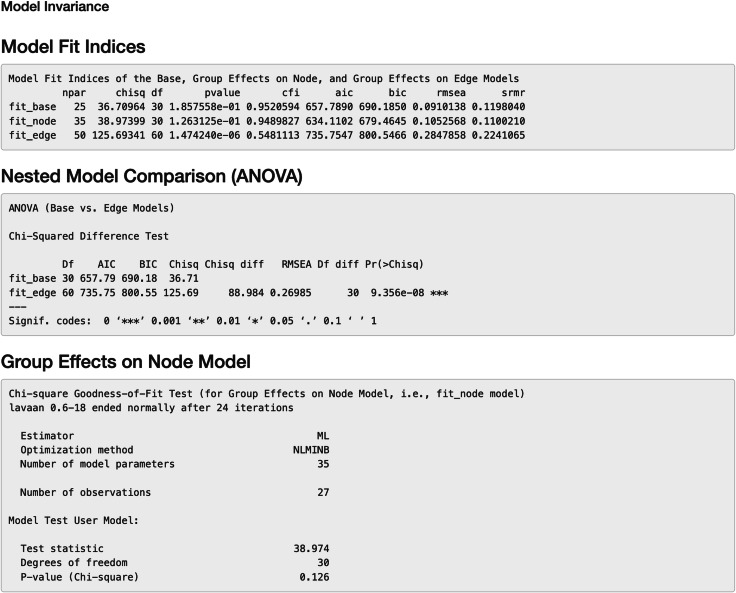
Model invariance result between two groups for the final model on Chagas Disease. The two groups were multiple sclerosis (MS) patients and healthy controls. “fit_base” refers to a model which does not consider group effects and assumes same patterns of relationships (*e.g.*, nodes, edges, and pathways) across groups. “fit_node” refers to a common model which assumes the node baselines (*e.g.*, baseline expression of genes or intercepts when all upstream regulators in the model are 0) are equal across groups. fit_edge” refers to a two-group model which assumes the strength or direction of relationships (*e.g.*, path coefficients and gene-gene interaction or edge weights) are equal across groups. npar = number of model parameters, chisq = chi-square goodness of fit test, df = degrees of freedom, pvalue = *p* value for chi-square goodness of fit test, “cfi” = comparative fit index, “aic” = Akaike information criterion, “bic” = Bayesian information criterion, “rmsea” = root mean square error of approximation, “srmr” = standardized root mean square residual, ANOVA = analysis of variance.

**Figure 8:**
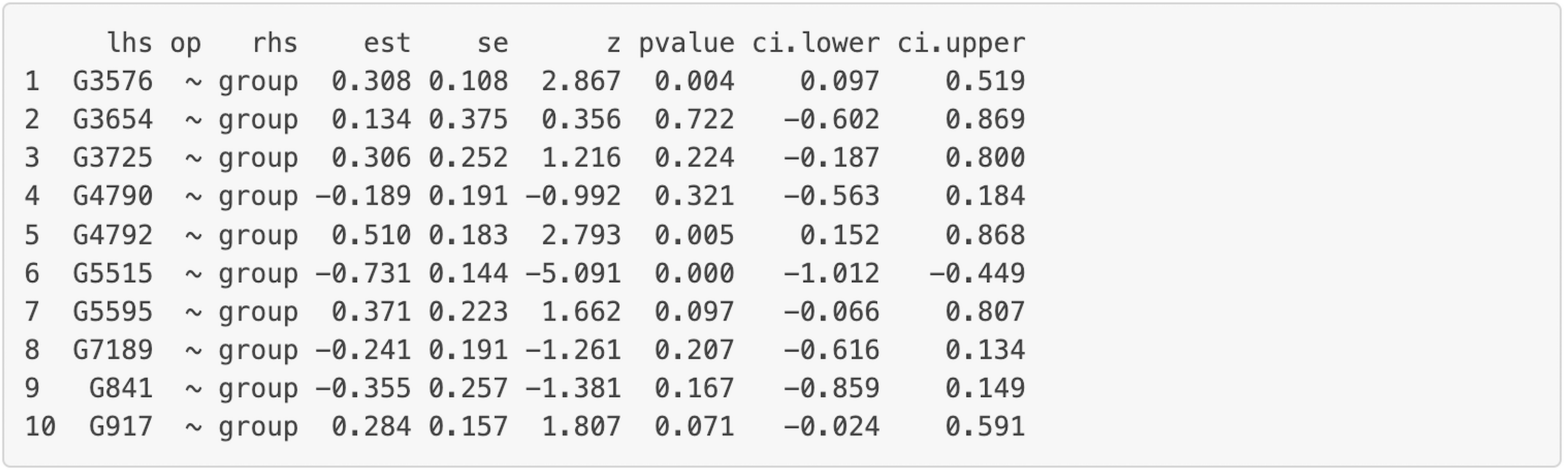
Node analysis result for the final model on Chagas disease pathway. “lhs” (left-hand side) denotes the dependent variable in the model. “op” = operator, “rhs” (right-hand side) represents the predictor variable. “est” represents estimated regression coefficient of the predictor variable on the dependent variable (*i.e.,* each gene’s expression or activity level). se = standard error of the estimated regression coefficient, z = standardized test statistic (*i.e., z* score) for the estimated regression coefficient. ci.lower = lower bound of the 95% confidence interval for the estimated regression coefficient, ci.upper = upper bound of the 95% confidence interval for the estimated regression coefficient. The symbol “∼” means “is regressed on”. group = 1 for patients with Multiple Sclerosis (MS), group = 0 for healthy controls.

**Figure 9:**
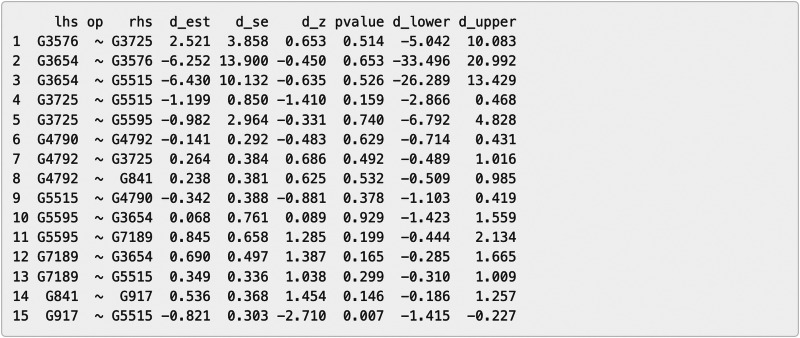
Part of the edge analysis result for the final model on Chagas Disease pathway. “lhs” (left-hand side) denotes the dependent variable in the model. “op” = operator, “rhs” (right-hand side) represents the predictor variable. d_est = path coefficient between two genes, quantifying the strength and direction of the gene influence, with a positive sign “+” denoting “activation” and a negative sign “−” denoting “inhibition” . d_se = standard error of the path coefficients, d_z = standardized *z*-score of the estimated path coefficient. d_lower = lower bound of the 95% confidence interval for the path coefficient, d_upper = upper bound of the 95% confidence interval for the path coefficient. The symbol “∼” means “is regressed on”.

Additionally, we can evaluate model invariance on edge and node concerning group membership in MS disease, which is similar to evaluating measurement invariance (Meredith, 1993; Vandenberg & Lance, 2000) on factor loadings and intercepts in a measurement model, respectively. We can evaluate the invariance based on model 6 by clicking “*Run Model Invariance*”. First, the output (see Fig. 7) showed model fit indices for the base model (*e.g.*, model 6, which did not consider group effects and assumed edge or node invariance), “group effects on edges” model (*i.e.,* a two-group model which examines group effects on edges), and “group effects on node” model (*i.e.,* a common model which examines group effects on nodes), respectively. For example, the RMSEA for the three models was .091, .105, and .285, respectively. Second, the analysis of variance (ANOVA) output comparing the “group effects on edge” model and the base model (see Fig. 7) showed that edge invariance was not supported for model 6 between the two groups, indicating that the weights for the gene-gene interactions between the two groups were not equal or “not statistically different”. See Fig. S3 for illustrated examples of models across groups. Third, the chi-square goodness of fit test on “group effects on node” model showed that node invariance was supported for this model (*p* > .05), meaning that the baseline gene expression levels for genes in the Chagas pathway were equal between the two groups, when all upstream regulators in the model were zero. If model invariance is violated, it is recommended that users run the SEM model related to group membership separately. By clicking “*Run Node Analysis*” and “*Run Edge Analysis*”, we can evaluate the strengths and directions of gene-gene interactions and the impact of group membership (see part of the results in Figs. 8 and 9).

## Discussion

### Model validation and causal interpretation

When performing SEM, we should consider the accuracy of research and the validity of results. We can examine configural, edge, and node invariances before performing group comparisons. For example, suppose an initial model for a specific pathway does not fit the data well and cannot be improved by adding paths, we may explore the consistency of the SEM structure regarding gene interactions across groups. Specifically, we can examine whether the same relationship patterns (*e.g.*, expressed genes or gene-gene interactions) hold across groups. We note that edge invariance is stricter and requires equal strength of relationships (*e.g.*, path coefficients or edge weights) across groups (Vandenberg & Lance, 2000). We can also examine node invariance to understand whether the baseline expression of genes is equal across groups. To control inflated Type I error from multiple comparisons, in the app, we used Brown’s combined goodness of fit test (Moskvina et al., 2011; Cinar & Viechtbauer, 2022) implemented in *SEMgraph* to evaluate whether nested SEM models (*e.g.*, base and “group effects on edge” models) fit the data equally well across groups. This approach complemented traditional likelihood ratio tests (LRTs) by aggregating evidence from multiple nested comparisons into a single statistical assessment (Cinar & Viechtbauer, 2022).

In addition, we can assess whether the coefficients of a specific pathway between groups differ statistically (*e.g.*, MS and “Chagas disease”) or investigate the relationships between different pathways. The evaluation enables researchers to examine gene regulation and expression differences between disease and control groups, facilitating our understanding of pathophysiology and treatment.

We clarify that the core purpose of SEM is to infer causal relationships rather than merely correlations. While correlation can indicate a relationship, SEM models how the activity of one gene directly influences the activity of another. The model uses path coefficients to quantify the strength and direction of these influences. A directed edge (A → B) indicates that gene A is an upstream regulator that directly affects the activity of gene B. The path coefficient quantifies the expected change in B’s activity resulting from a change in A’s activity. This influence does not have to be a direct and positive correlation at the transcript level.

### Considerations for SEM data in biological applications

The SEM in this paper can use data from gene expression microarrays and incorporate information from other sources to build and refine the initial model. The initial model in the demonstration uses curated biological pathways from databases such as KEGG, which provide information about various gene relationships, including regulatory relationships, protein–protein interactions, and metabolic pathways. According to Pepe & Grassi (2014), the model is further refined by identifying the shortest paths between differentially expressed genes (DEGs), which tailors a model specific to the observed changes in the gene expression data. Genes not differentially expressed but part of the shortest path are grouped into Protein Information Resource (PIR) superfamilies based on evolutionary relationships (2014), potentially highlighting standard functions or regulatory mechanisms.

Databases like STRING can provide information on known and predicted protein-protein interactions and functional associations. That information can be applied to inform model modification by adding new directed or bi-directed edges based on biological evidence.

### Phosphorylation and causal inference in structural equation modeling of transcriptomic data

Phosphorylation-mediated regulation presents a unique challenge in transcriptomic analyses (Hunter, 2000; Olsen & Mann, 2013), as the causal influence of one gene (gene *A*) on another (gene *B*) may not correlate strongly with their respective RNA levels. For example, when the protein product of gene *A* phosphorylates gene *B*’s protein: while increased transcription of *A* may elevate *A* protein levels and subsequently modify gene B’s activity, gene *B*’s mRNA levels may remain unchanged. Structural equation modeling addresses this limitation by detecting consistent directional relationships between genes, even in cases where transcript abundances appear independent. SEM achieves this by estimating path coefficients that reflect the net directional effect of gene *A* on gene *B*, incorporating both direct (*e.g.*, phosphorylation-mediated) and indirect regulatory mechanisms. The model assesses whether systematic covariation exists between changes in *A*’s expression and downstream functional impacts on *B*’s activity, whether measured directly through activity measurements (*e.g.*, functional assays) or inferred through proxy gene expression patterns.

Model evaluation involves rigorous testing of proposed relationships. A poorly fitting edge (A → B) indicates a mismatch between the modeled relationship and the underlying biological mechanisms. Researchers may refine the model by adding or removing edges based on modification indices and biological plausibility. In addition, multiple-group analysis enables the comparison of model parameters across experimental conditions, revealing context-specific differences in regulatory strength and direction that may reflect condition-dependent phosphorylation states or other post-translational modifications.

This approach provides particular value in cases where post-translational regulation decouples protein activity from transcript abundance. By focusing on systematic patterns of covariation across multiple measurements, SEM can infer causal relationships that would be obscured by examining RNA correlations alone. However, the validity of such inferences depends critically on iterative model refinement and integration of complementary biological evidence.

## Conclusion

This study presents ShinyDegSEM, an interactive application that implements a pathway-constrained SEM framework to analyze gene regulatory networks. By incorporating prior pathway knowledge (*e.g.*, KEGG pathways) to guide model structure, the app enables researchers to estimate direct regulatory effects between observed gene expression levels and compare these relationships across experimental or clinical conditions. The tool’s user-friendly interface democratizes advanced statistical modeling, eliminating the need for specialized coding expertise and bridging the gap between computational biology and experimental, clinical, or population research.

We have currently demonstrated the use of ShinyDegSEM modeling to investigate gene interactions within individual pathways, providing a biologically interpretable framework for generating and validating hypotheses. The current version performs a log2 transformation on the microarray data for normalization within the app, which also accepts log2-transformed single-cell RNA-seq counts (matching microarray preprocessing) and can apply the SEM model on transformed RNA-seq data (van Montfort, Mooijaart & Meijerink, 2009). In the next version, we will integrate the SCTransform normalization method (implemented in the *sctransform* R package or Seurat toolkit; Hafemeister & Satija, 2019) for single-cell RNA-seq and the variance-stabilizing transformation (VST) from *DESeq2* (Love, Huber & Anders, 2014) for bulk RNA-seq to natively address zero-inflation and overdispersion. In addition, we plan to maintain and expand the application’s functionality to investigate longitudinal gene data or integrate multi-omics data, further enhancing its utility for dynamic changes or systems-level analyses. By combining accessibility with rigorous statistical methods, ShinyDegSEM has the potential to accelerate discoveries in gene regulatory research and foster interdisciplinary collaboration.

##  Supplemental Information

10.7717/peerj.20033/supp-1 Supplemental Information 1 Explanations on gene expression data method and structural equation modeling

## List of Abbreviations

App: Application
DEGs: Differentially Expressed Genes
FC: Fold-change
FTLD-U: Frontotemporal Lobar Degeneration with Ubiquitinated Inclusions
GEO: Gene Expression Omnibus
GOF: Goodness of Fit
GWAS: Genome-wide Association Studies
KEGG: Kyoto Encyclopedia of Genes and Genomes
LRTs: Likelihood Ratio Tests
MI: Modification Indices
ML: Maximum Likelihood
MS: Multiple Sclerosis
NCBI: National Center for Biotechnology Information
Non-DEGs: Non-differentially Expressed Genes
PBMC: Peripheral Blood Mononuclear Cells
PIR: Protein Information Resource
PTMs: Post-translational modifications
QRT-PCR: Quantitative Real-time Polymerase Chain Reaction
QTL: Quantitative Trait Loci
QTLnet: Quantitative Trait Loci (QTL)-driven Phenotype Network Method
RMSEA: Root Mean Square Error of Approximation
RNA-seq: RNA-sequencing
SAM: Significance Analysis of Microarrays
SCZ: Schizophrenia
SEM: Structural Equation Modeling
SM: Supplementary Materials
SML: Sparsity-aware Maximum Likelihood
SPIA: Signaling Pathway Impact Analysis
SRMR: Standardized Root Mean Square Residual
STRING: Search Tool for the Retrieval of Interacting Genes/Proteins Database

## Additional Information and Declarations

### Data Availability

The following information was supplied regarding data availability: The datasets analyzed in this study are available in the Gene Expression Omnibus database: GSE21942.
