# Supplementary material for: ShinyDegSEM: an interactive application for pathway perturbation analysis in gene expression studies via structural equation modeling
Source: PeerJ. 2025 Oct 10;13:e20033. doi: 10.7717/peerj.20033 (PMC12517285; doi:10.7717/peerj.20033)
Supplement: Supplemental Information 1 [file peerj-13-20033-s001.pdf]

Supplementary Materials

Differentially Expressed Genes (DEGs): Genes that exhibit statistically significant changes in their expression levels when comparing two or more biological conditions. These changes can be up-regulation (increased expression) or down-regulation (decreased expression).
Signaling Pathway Impact Analysis (SPIA): A statistical method used to identify perturbed biological pathways in gene expression data. It combines information about gene expression changes with prior knowledge of pathway structure.
Structural Equation Modeling (SEM): A statistical approach for modeling the causal relationships among observed and latent variables. In the context of gene expression data, SEM is used to infer the direction and strength of gene-gene interactions within perturbed pathways (Pepe & Grassi, 2014). *Conventional SEM* typically includes two main components: the measurement and the structural model (Pepe & Grassi, 2014; Kline, 2023).

(i) Measurement Model:

This model relates observed variables (which we can directly measure, such as gene expression products like RNA or protein levels) to latent variables (which we cannot measure directly, such as genes regulatory activities, hidden biological states, or unmeasured pathways that shape phenotypes).
Mathematical Formula:

$$\boldsymbol{X} = \boldsymbol{\Lambda}\boldsymbol{\xi} + \boldsymbol{\varepsilon}$$

Where $\boldsymbol{X}$ is a vector of observed variables. $\boldsymbol{\Lambda}$ (Lambda) is a matrix of factor loadings, which quantify the strength of the relationship between each observed variable and each latent variable. $\boldsymbol{\xi}$ (Xi) is a vector of latent variables or factors. $\boldsymbol{\varepsilon}$ is a vector of measurement errors.
This equation expresses that each observed variable X is a linear combination of latent variables, ξ, plus measurement error ε (Epsilon).
Purpose: The measurement model helps us understand how well our observed variables measure the underlying latent constructs.

(ii) Structural Model:
This model specifies the relationships between latent variables.
Mathematical Formula:

$$\boldsymbol{\eta} = \boldsymbol{B}\boldsymbol{\eta} + \boldsymbol{\Gamma}\boldsymbol{\xi} + \boldsymbol{\zeta}$$

Where $\boldsymbol{\eta}$ (Eta) is a vector of endogenous latent variables (influenced by other variables in the model). $\boldsymbol{B}$ is a matrix of path coefficients that describe the effect of endogenous latent variables on each other. $\boldsymbol{\Gamma}$ (Gamma) is a matrix of path coefficients that describe the effect of exogenous latent variables $\boldsymbol{\xi}$ on endogenous latent variables $\boldsymbol{\eta}$. $\boldsymbol{\xi}$ is a vector of latent variables or factors. $\boldsymbol{\zeta}$ (Zeta) is a vector of structural errors.

This equation says that each endogenous latent variable η is a linear combination of other endogenous variables $\boldsymbol{\eta}$, exogenous variables $\boldsymbol{\xi}$, plus a structural error term.

Purpose: The structural model tests hypotheses about the causal relationships between latent constructs.

Focusing on Observed Variables in this Paper: A Converted SEM

The SEM approach used in the application differs from the conventional approach. In this paper, we used a converted version of SEM where:

- **No Latent Variables in the Measurement Model**: The paper examined the relationships between observed gene expression data directly. Therefore, there is no measurement model needed, and the analysis is converted.
- **Focusing on Direct Relationships**: Instead of analyzing relationships between latent variables, the focus shifts to the relationships between the observed variables (gene expression levels) and unobserved factors.
- **The system of linear equations** used in the paper is as follows:

Equation 1 (for influence of parent genes):

$$Y_i = \Sigma_{j \in pa(i)} \beta_{ij} Y_j + U_i$$

Where Y_i is the expression level of gene i (observed). pa(i) is the set of parent genes directly influencing gene i (observed). β_{ij} is the path coefficient (influence of gene j on gene i). U_i is an unmeasured variable, which is similar to a structural error term in conventional SEM.

Equation 2 (for covariance of unmeasured variables):

$$cov(U_i, U_j) = \begin{cases} \psi_{ij} & if\ i = j\ or\ j \in sib(i) \\ 0 & Otherwise \end{cases}$$

Where $cov(U_i, U_j)$ is the covariance between unmeasured variables. ψ_{ij} is the covariance parameter (relationship between two unmeasured variables). $sib(i)$ is the set of "sibling" genes where the unmeasured factors may be correlated.

- **Observed variables**: The model directly uses observed gene expression levels as variables.
- **Path diagram**: These equations are represented in a path diagram, which is a visual tool, with both directed (→) and bi-directed (↔) edges to represent relationships between genes and their unmeasured factors. **Directed edges** represent the direct influence of one gene on another through path coefficients (β_{ij}). **Bi-directed edges** represent the correlation between unmeasured variables (ψ_{ij}), indicating the presence of a hidden common cause behind variations in gene expression.

- **Modified SEM framework:** Our modified SEM estimates direct regulatory effects (β_{ij}) between observed gene expression levels, with the model structure constrained by prior pathway knowledge (e.g., KEGG). Sibling genes (i.e., sib(i), genes co-regulated by shared unmeasured factors) are identified either from pathway annotations or inferred from residual correlations. Bi-directed edges (ψ_{ij}) represent shared unmeasured factors and are jointly estimated with β_{ij} via maximum likelihood.

Pathway Analysis: Identifying and analyzing the interactions between genes and proteins within biological pathways. The analysis helps us understand how changes in gene expression can affect cellular processes and disease states.

Gene Expression Microarrays: A high-throughput technology used to measure the expression levels of thousands of genes simultaneously. The resulting data is used to identify gene expression patterns associated with specific biological conditions or diseases.

KEGG Database: Kyoto Encyclopedia of Genes and Genomes, a comprehensive database of biological pathways, gene functions, and other molecular networks. It is commonly used in pathway analysis and gene network reconstruction.

Tests and Model fit

(i) Chi-square χ^2 Test

- What it is: Assesses the difference between the observed covariance matrix and the model-implied covariance matrix.
- How it's calculated: $\chi^2 = -2\log LR = -2[\log L(\Sigma(\theta)) - \log L(\Sigma_0)]$, where LR = likelihood ratio, $\Sigma(\theta)$ = model implied covariance matrix, Σ_0 = true population covariance matrix.
- Interpretation:
  - Non-significant p-value ($p > .05$): Good fit.
  - Significant p-value ($p < .05$): Poor fit.

(ii) Root Mean Square Error of Approximation (RMSEA)

- What it is: Measures the discrepancy between the hypothesized model and the population covariance matrix, adjusting for model complexity.
- Formula: $\text{RMSEA} = \sqrt{\frac{\max(0,\ \chi^2 - df)}{df*(n-1)}}$
- Interpretation:
  - $< .06$: Acceptable fit.
  - $> .06$: Poor fit.

(iii) Standardized Root Mean Square Residual (SRMR)

- What it is: Measure of the average difference between the observed and predicted correlations.
- Formula: $\text{SRMR} = \frac{\sum_{i=1}^{p}\sum_{j=i+1}^{p}(s_{ij} - \widehat{\sigma_{ij}})^2}{p(p+1)/2}$,

  Where p = the number of genes, i = ID for gene i, j = ID for gene j, s_{ji} = observed covariance between genes i and j. $\widehat{\sigma_{ij}}$ = model estimated covariance between genes i and j.
- Interpretation:
  - $< .08$: Acceptable fit.
  - $> .08$: Poor fit.

Figure 1

Directed Edge Between Genes Y_1 and Y_2

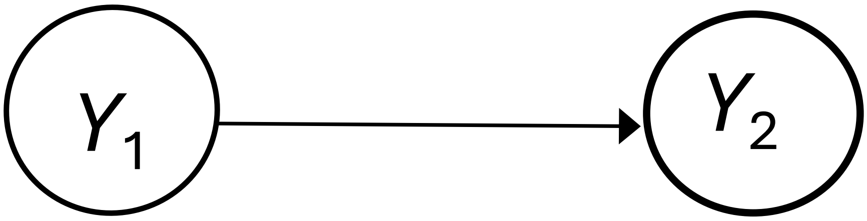

Note. Circles denote nodes (i.e., genes Y_1 and Y_2), respectively. The symbol → denotes a directed edge, indicating Y_1 (upstream regulator) regulating Y_2.

Figure 2

Bidirected Edge Between Genes Y_1 and Y_2

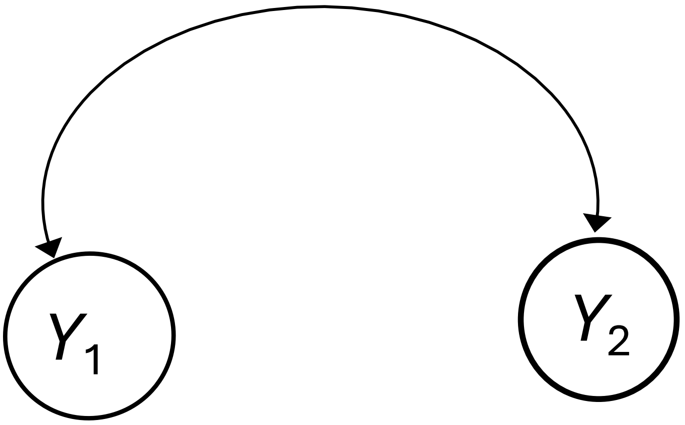

Note. Circles denote nodes (i.e., genes Y_1 and Y_2), respectively. The symbol ↔ denotes a bidirected edge, indicating covariance between two genes due to an unmeasured common cause (e.g., latent upstream regulators or shared environmental factors).

Figure 3

Models for Different Levels of Model Invariance Between Group 1 and Group 2

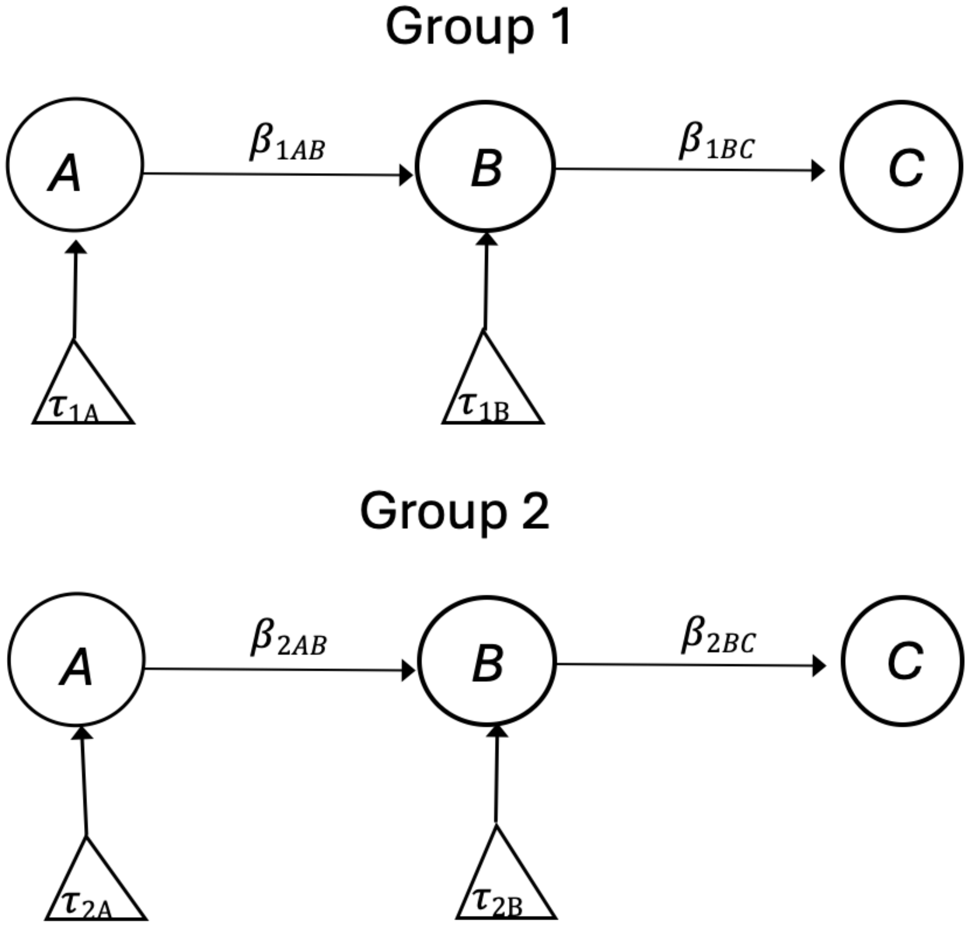

Note. Circles denote nodes (i.e., genes *A, B,* and *C*), respectively. "→" symbol between genes A and B refer to an edge between them. β_{1AB} = path coefficient (i.e., edge strength) between genes A and B for group 1, β_{1Bc} = path coefficient between genes B and C for group 1, τ_{1A} = intercept for using gene A predicting gene B for group 1, τ_{1B} = intercept for using gene B predicting gene C for group 1. β_{2AB} = path coefficient between genes A and B for group 2, β_{2Bc} = path coefficient between genes B and C for group 2, τ_{2A} = intercept for using gene A predicting gene B for group 2, τ_{2B} = intercept for using gene B predicting gene C for group 2. "**fit_base**" refers to a model that does not consider group effects and assumes the same patterns of relationships (e.g., nodes, edges, and pathways) across groups. "**fit_node**" refers to a common model that assumes the node baselines (e.g., baseline expression of genes or intercepts when all upstream regulators in the model are 0) are equal across groups (i.e., $\tau_{1A} = \tau_{2A}$ and $\tau_{1B} = \tau_{2B}$). "**fit_edge**" refers to a two-group model that assumes the strength or direction of relationships (e.g., path coeûcients and gene-gene interaction or edge weights) are equal across groups (i.e., $\beta_{1AB} = \beta_{2AB}$ and $\beta_{1BC} = \beta_{2BC}$)
